# Parental Involvement in Adolescent Psychological Interventions: A Meta-analysis 

**DOI:** 10.1007/s10567-024-00481-8

**Published:** 2024-05-15

**Authors:** Abigail E. Pine, Mary G. Baumann, Gabriella Modugno, Bruce E. Compas

**Affiliations:** Department of Psychology and Human Development, Vanderbilt University, Peabody 552, 230 Appleton Place, Nashville, TN 37203 USA

**Keywords:** Adolescence, Intervention, Parents, meta-analysis

## Abstract

Psychological interventions for adolescents have shown mixed efficacy, and including parents in interventions may be an important avenue to improve treatment outcomes. Evidence from meta-analyses examining the role of parents in interventions for youth is inconsistent and has typically combined findings for both children and adolescents together. No prior meta-analysis has examined the specific role of parents in adolescent interventions as compared with interventions focused solely on adolescents across several disorders. To address this gap, systematic literature reviews were conducted utilizing a combination of searches among keywords including (parent * OR *family*) AND (*intervention* OR therap * OR *treatment* OR prevent*) AND (adolescen*). Inclusion criteria were (1) a randomized controlled trial of an individual psychological intervention compared to the same intervention with a parental component, and (2) adolescents must have at least current symptoms or risk to be included. Literature searches identified 20 trials (*N* = 1251). Summary statistics suggested that interventions involving parents in treatment have a significantly greater impact on adolescent psychopathology when compared to interventions that targeted adolescents alone (*g* = − 0.18, *p* < .01, 95% CI [− 0.30, − 0.07]). Examination with symptom type (internalizing or externalizing) as a moderator found that the significant difference remained for externalizing (*g* = − 0.20, *p* = .01, 95% CI [− 0.35, − 0.05]) but not internalizing psychopathology (*p* = .11). Findings provide evidence of the importance of including parents in adolescent therapy, particularly for externalizing problems.

## Introduction

Interventions to effectively treat psychological disorders in adolescence are a high priority for clinical psychological science. A challenge for the field is to determine if these interventions are best delivered individually to adolescents or if there is value added to involve parents in the treatment of adolescent disorders. The current study addresses this need by presenting the results of a meta-analysis comparing individually focused interventions for adolescents to interventions that include a parent intervention in addition to individual treatment. This introduction is presented in three sections. The first section describes features of developmental psychopathology with a focus on adolescence. This includes overall rates of disorders, the impact of psychopathology during adolescence, and important features of development that may contribute to risk. Next, the complex relations between family processes and adolescent psychopathology are described. Finally, results from clinical trials that attempt to improve treatment response in adolescent psychopathology by involving parents in intervention are reviewed. The rationale for a quantitative meta-analysis is provided, focused on effects of augmenting response to psychotherapy in adolescents by including parents in treatment.

### Adolescence as an Important Developmental Period

Adolescence is characterized as a period of significant biological and psychosocial change, coinciding with an increased risk for the development of psychopathology (Costello et al., 2011; Merikangas et al., 2010; Steinberg & Morris, 2001). Data from a population-based, prospective longitudinal study across development suggest that one in three youth will have at least one mental health disorder by age 16, with a marked increase in rates of depression, social phobia, and substance use occurring during adolescence (Costello et al., 2003). Cumulative prevalence rates are even more striking as disorders continue to increase into late adolescence and emerging adulthood, suggesting that as many as 61% will meet criteria for a disorder by age 21 (Copeland et al., 2011). Estimates vary based upon study design and type of assessments conducted (Costello et al., 2005; Duffy et al., 2023; Moffitt et al., 2010), but researchers agree that psychopathology in adolescence is a significant public health concern.

Psychopathology in adolescence is associated with significant psychosocial impairment (Clayborne et al., 2019; Kajastus et al., 2023; Shapero et al., 2013), as well as risk for problems into adulthood (Copeland et al., 2015). Specifically, psychopathology in adolescence has been linked to poor school performance (Kajastus et al., 2023), future unemployment (Clayborne et al., 2019), and peer victimization (Shapero et al., 2013). Further, mental health difficulties in childhood and adolescence are associated with a 2- to 6-fold increase in risk for diagnoses (Hofstra et al., 2002) and six times higher odds of adverse outcomes in adulthood (Copeland et al., 2015). Given the high prevalence and long-term impact, effective interventions targeting psychopathology in adolescence are critical.

### Family Processes and Adolescent Psychopathology

There are well-established links between family processes and youth psychopathology (e.g., King et al., 2016; McKee et al., 2008; Velleman et al., 2005; Yap et al., 2014). Prior studies have emphasized the importance of parental warmth (Rothenberg et al., 2020; Yap et al., 2014) and authoritative parenting practices (King et al., 2016; Morris et al., 2021) in decreasing risk for internalizing and externalizing psychopathology among adolescents. Although some insights about family processes and psychopathology can be gleaned from work involving children, significant neurobiological and psychosocial changes occur during adolescence (Hostinar et al., 2015; Suleiman & Dahl, 2019). These changes have unique implications for understanding family processes and risk during this developmental time. These changes include the onset of puberty with associated alterations in brain development and multiple changes in social relationships as well as effects on interpersonal functioning, including with parents.

The role of parents changes as youth enter puberty and seek growing autonomy and independence. Adolescents spend increasing amounts of time with peers, and parents must grapple with navigating the importance of promoting youth autonomy while maintaining adequate supervision and connectedness (Morris et al., 2021). This change often, although not always (Steinberg & Morris, 2001), generates tension in parent–adolescent communication and interactions (Steinberg & Silk, 2002) as both parents and adolescents realign roles and expectations. Therefore, adolescence is a developmental period characterized by changes in interactions with parents and presents parents with unique challenges that are not encountered during interactions with younger children.

Despite these changes, several pivotal studies have pointed to the protective role of parents in altering risk trajectories for psychopathology during adolescence, even as the importance of peer support increases (Anderson et al., 2015; Hazel et al., 2014; Herres & Kobak, 2015; Manczak et al., 2019; Quiroga et al., 2017; Van der Giessen et al., 2014). In fact, evidence suggests that parental support may have a buffering effect on risk for psychopathology among adolescents experiencing peer difficulties (Hazel et al., 2014; Herres & Kobak, 2015), romantic stress (Anderson et al., 2015), and exposure to violence (Quiroga et al., 2017). Such significant findings lay a strong foundation for the importance of parents in adolescent psychopathology.

### Psychological Interventions with Adolescents

When considering the developmental considerations described above, there is reason to suggest that the role of parents within adolescent interventions may differ from how they are involved with children. Adolescent interventions stem from “downward adaptations of adult treatments or upward adaptations of child treatments” (Weisz & Hawley, 2002). However, adolescence is a unique developmental time period, and involving parents in interventions may bring novel challenges and ethical dilemmas (Bolton Oetzel & Scherer, 2003; Duncan & Sawyer, 2010; Meade & Slesnick, 2002). For example, therapists may struggle to decide when to break confidentiality in situations of risk when working with maturing adolescents who still live within their parents’ household. As such, therapists must demonstrate particular care when including parents in adolescent therapy.

Adolescents strive for autonomy, and independence may be particularly important for them within the context of a therapeutic relationship. However, this has the potential to create difficulties for therapists trying to respect the autonomy and confidentiality of an adolescent while also recognizing the influence that parents may have, the legal responsibilities parents have for the welfare of their adolescent children, as well as the importance of including them in high-risk situations. Therapists must be attuned to balancing both the dynamics of a “working alliance” with parents at the same time as a “therapeutic alliance” with adolescents (Schimel, 1974). Relations among therapists, parents, and adolescents may be further complicated, as findings suggest that more than 75% of child–parent–therapist triads fail to agree on the main focus of treatment (Hawley & Weisz, 2003). There is reason to believe that involving parents in adolescent therapy is beneficial, but questions remain about the best way to go about doing so.

### Parental Involvement in Psychological Treatments for Adolescents

The developmental and cognitive considerations of pre-adolescent children often require parents to be included in many if not all aspects of interventions (Comer et al., 2019; Grave & Blissett, 2004). This is in contrast to adolescents who begin to develop the complex social-cognitive skills (Crone & Dahl, 2012) required to engage in individual and group evidence-based interventions (Frankel et al., 2012). As a result, larger proportions of time during therapy may be spent with adolescents and the therapist alone, as compared to children, where more time may jointly involve the child, parent, and therapist. Nevertheless, there still can be a role for work with parents. Although the importance of independence, autonomy, and peer relationships increases during adolescence, parents remain an essential influence throughout this developmental time period (Steinberg & Morris, 2001). Moreover, current individually focused interventions for adolescents are not effective for all youth (Weisz et al., 2017), and thus, increasing parent involvement in adolescent interventions may be an important pathway to improve efficacy of interventions.

Existing reviews and meta-analyses have examined the benefits of involving parents in interventions among both children and adolescents (Beelmann et al., 2023; Dippel et al., 2022; Dowell & Ogles, 2010; Peris et al., 2021; Sandler et al., 2015; Thulin et al., 2014). Findings are inconsistent as to the potential benefit of parent-involved interventions (Dippel et al., 2022; Peris et al., 2021; Thulin et al., 2014). Some of the variability in findings may have arisen from effects of moderators, including intervention type (Dowell & Ogles, 2010) and age of youth (Beelmann et al., 2023). Dowell and Ogles (2010) included studies across diagnoses in a direct comparison of an individual child treatment to either family therapy or a combined individual and parent intervention and found that parent/family treatments performed better than individual child treatments (*d* = 0.27), particularly when non-cognitive-behavioral therapy (CBT) individual treatments were utilized. In addition, among meta-analyses that have examined the impact of age, some have not found age to be a significant moderator of treatment efficacy (Dowell & Ogles, 2010), while others have found a small trend for younger children evidencing greater benefits from parent-involved treatment (Beelmann et al., 2023).

There are few meta-analyses (Couturier et al., 2013; Vermeulen-Smit et al., 2015) published on the role of parental involvement with exclusively adolescent samples, and none have examined the impact of parental involvement across different diagnoses. While there have been several narrative reviews published on the role of parental involvement with adolescent interventions (Cardy et al., 2020; Dardas et al., 2018; Kuntsche & Kuntsche, 2016; Medlow et al., 2016; Newton et al., 2017), the lack of quantitative data limits the conclusions that can be drawn from such studies. Findings from two existing meta-analyses examining the efficacy of family interventions in the prevention of adolescent drug use (Vermeulen-Smit et al., 2015) and treatment of adolescents with eating disorders (Couturier et al., 2013) yielded inconclusive findings and vary based upon disorder assessed. No prior meta-analysis to the authors’ knowledge has examined the role of parents in adolescent interventions across several diagnoses.

### Importance of Study Design

Additional variability in findings examining the role of parents in youth interventions may result from the designs used in studies in this area. For example, many studies have compared parent-involved interventions to a no treatment or control condition (Cardamone-Breen et al., 2018; Chaplin et al., 2021; Connell & Dishion, 2008; Diamond et al., 2010; Kogan et al., 2016; Mason & Spoth, 2012), while others have involved comparisons to a different type of individual intervention (Brent et al., 1997; Dakof et al., 2015; Lock et al., 2010; Slesnick et al., 2013; van der Pol et al., 2018). Similar to evidence-based interventions more broadly (Weisz et al., 2017), parent-involved interventions have been found to be significantly more beneficial when compared to no treatment or waiting list control conditions (Chaplin et al., 2021; Kogan et al., 2016). Findings are less clear when compared to individual interventions (Lock et al., 2010; Slesnick et al., 2013). There is evidence to suggest a benefit to including parents in adolescent interventions when compared to a control condition, but there is limited clarity as to what extent parental involvement may be beneficial above and beyond an active individual intervention. The ideal randomized controlled trial would assess the efficacy of a parent-involved intervention when compared to an individual intervention.

### Current Study

The current meta-analysis aims to clarify ambiguity in the literature by including randomized controlled trial designs whereby an individual treatment is compared to the *same* individual treatment with an added parental involvement component. This design is intended to decipher any benefit of parental involvement above and beyond individual treatment. Although it is likely that the importance of parents in interventions differs based upon diagnosis, all parent-involved work with adolescents must navigate the unique psychosocial stressors of changes in autonomy alongside pubertal developmental and increased risk for psychopathology. This paper serves as a preliminary review of the current literature related to this question, so all available disorder groups are included. It is hypothesized that compared to individual treatment, individual treatment with an added parental involvement component will result in significantly better therapeutic benefit for adolescents.

## Method

**Fig. 1:**
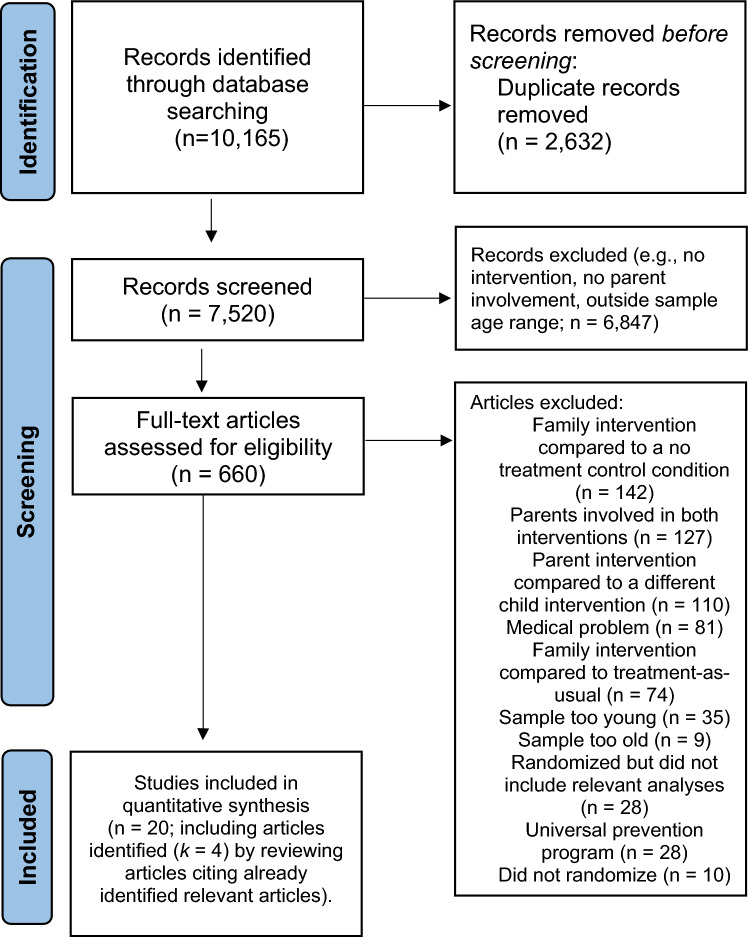
Prisma flow diagram

The current review was conducted in accordance with the Preferred Reporting Items for Systematic Reviews and Meta-Analyses (PRISMA) guidelines (see Fig. 1; Page et al., 2021). The literature searches utilized the PsycINFO database to capture a wide variety of adolescent prevention and treatment interventions for various diagnoses in which parents may have been included. Articles, including peer-reviewed manuscripts and unpublished dissertations, were identified from 1934 through August 23rd, 2022, and then further updated as of July 1st, 2023. Searches included combinations among keywords (parent* OR *family*) AND (*intervention* OR therap* OR *treatment* OR prevent*) AND (adolescen*). The Covidence program (*Covidence Systematic Review Software*) was then used to remove duplicates (*k* = 2632) and systematically sort through articles. In the total included papers, corresponding authors were contacted if the necessary data were not reported (*k* = 3).

Inclusion criteria for the current review were: (1) the age range in studies only included adolescents, defined as the second decade of life (ages 10–19 years old; Lerner & Steinberg, 2004, World Health Organization); (2) study design must include a randomized controlled trial whereby an individual psychological intervention was compared to the same individual intervention with the addition of a parental component; (3) the target of the intervention is a mental health diagnosis such that psychotherapy trials within the context of a medical condition (e.g., asthma, cancer, or obesity) were excluded; (4) adolescents involved in the trial must have at least current symptoms or be at-risk for a disorder to be included; (5) psychopathology outcome variables were measured in both groups at least one time following the conclusion of treatment; and (6) articles must be published in English.

### Study Selection

Articles (*N* = 7533) were initially screened to identify those that included randomized controlled trial designs and any form of parent-involved treatment with adolescents. Parental involvement was defined as active participation of the parent within the intervention, including psychoeducation and parenting or communication skills acquisition. Interventions whereby parents were simply updated about their adolescent’s progress or treatment plans were not sufficient to be classified as a parent-involved intervention. The number of sessions parents were involved in varied by study. There was no minimum number of parent-involved sessions required to be included in the meta-analysis, and each included intervention comprised at least two sessions. This left 660 articles to be assessed for eligibility. Reasons for exclusion included failure to meet the necessary study design, such as not randomizing families to groups or utilizing a different intervention as a comparison condition. Articles were narrowed down to only include randomized controlled trial designs whereby a parent/family intervention was compared to an individual intervention, excluding interventions that included parents that were compared to a no treatment control condition (*k* = 142) or treatment as usual (*k* = 74). A significant number of studies compared a parent intervention to a different individual child intervention (e.g., family-based treatment vs. individual CBT; *k* = 110) or a different intervention that also included parents (*k* = 127). These were also removed as comparison to a different intervention orientation, or an intervention that already contained some level of parental involvement, produced more noise and ambiguity beyond assessing the pure question of what the benefit of involving parents may be. Additionally, as noted above, studies that focused on a health problem (e.g., obesity; *k* = 81) or a universal preventive intervention (*k* = 28) were excluded given the primary interest in parent involvement with youth with symptoms of psychopathology. Several studies (*k* = 35) included a sample age range with both children and adolescents (e.g., 7–14 years old), and these were also excluded given the primary focus of this meta-analysis is on adolescence. More articles were identified (*k* = 4) by reviewing those citing already identified relevant articles. The final sample of articles was reviewed by the first author to ensure that the primary paper from each included trial was represented. Included dissertations and peer-reviewed articles were carefully reviewed to ensure the prevention of duplicates. A subset (20%) of the full-text articles screened for eligibility were double-coded to assess for inter-rater reliability. Rater agreement across articles reviewed was 96%, κ = 0.80. If raters disagreed about inclusion, they discussed until consensus was obtained.

### Data Analysis

Quantitative analyses were conducted in Comprehensive Meta-Analysis (CMA) program version 4 (Borenstein et al., 2022). Random effects models were utilized (Borenstein et al., 2010), as it was assumed that effect sizes will vary based upon different study characteristics. Several of the included studies reported many relevant outcomes variables, so the primary, continuous measure of adolescent psychopathology at the closest time point to end of treatment was utilized when available. If two or more variables met this criterion, the mean of the scores for these measures was utilized. Where possible, the standardized mean difference between the individual intervention and parents-included intervention was calculated and used as the effect size. Four studies (Barrett et al., 2001; Bernal et al., 2019; Dennis et al., 2004; Reuland & Teachman, 2014) only reported relevant dichotomous outcomes, such as whether youth still met criteria for a diagnosis following the intervention, and therefore, effect size was computed by calculating the log odds ratio for these data. Different effect sizes among studies were compared after being computed into an unbiased estimate, Hedges’ g (Hedges, 1981). Heterogeneity was examined with *Q* and *I*^*2*^ statistics, and publication bias was conducted by visually inspecting funnel plots and calculating Egger’s tests (Egger et al., 1997). Sensitivity analyses were performed through the CMA program, whereby effect sizes were systematically recalculated as each individual study was removed. Past meta-analyses have assessed parental involvement separately for different disorders (Couturier et al., 2013; Vermeulen-Smit et al., 2015), so symptom type (internalizing or externalizing) was included as a moderator. Both ADHD and substance use outcomes were coded as externalizing given their connections in dimensional models of psychopathology (Krueger et al., 2021). Additional moderators tested included number of sessions parents were involved in, outcome type, outcome assessment timeframe, age, and study quality, assessed with Jadad criteria (Jadad et al., 1996).

## Results

### Study Characteristics

All searches yielded a total of 20 trials meeting inclusion criteria (*N* = 2270 participants). The average age of participants in the included studies was 14.67 years, the average percentage of females in the study was 51.8%, and the average sample size was 113.5 families. Data were extracted from each study, including study design, population, age range, follow-up time point, relevant outcomes included in the meta-analysis, and results. These data are presented further in Table 1. Table 2 details information about the individual and parent-involved interventions. Relevant effect size data were not able to be obtained for three studies (Hardway et al., 2015; Hooven et al., 2012; Spirito et al., 2015), so these were not included in quantitative analyses.

**Table 1:** Characteristics of studies included in meta-analysis

Trial	Design	Sample	Age range (years)	Time point included in meta-analysis	Internalizing or externalizing	Primary outcome(s) included in meta-analysis	Results
Bernal et al. (2019)	CBT vs. CBT + Taller de Educacion Psicologica para Padres y Madres	*N* = 121 with MDD diagnosis	13-17.5	Post-treatment	Internalizing	Number in remission based on MDD diagnosis with Diagnostic Interview Schedule for Children	No significant differences between groups
Bogle, 2007 dissertation	Challenging Horizons Program vs. Challenging Horizons Program + Academic Skills Building workshops	*N* = 34 with ADHD diagnosis or impairment in functioning	11–13	Post-treatment	Externalizing	Conner’s Global Index parent report	No significant differences between groups (*d* = 0.06)
Dennis et al. (2004)	Motivational-Enhancement Therapy & CBT vs. Motivational-Enhancement Therapy & CBT + Family Support Network	*N* = 198 with one or more DSM-IV criteria for cannabis use or dependence	12–18	12 month	Externalizing	Number in recovery, defined as living in the community and reporting no substance use, abuse, or dependence problems in the past month	No significant differences between groups
Clarke et al. (1999)	CBT vs. CBT + parent group	*N* = 96 with DSM-III-R diagnosis of MDD or dysthymia	14–18	Post-treatment	Internalizing	Hamilton Depression Rating Scale, Beck Depression Inventory	No significant differences between groups
Dishion & Andrews (1995)	Parent focus vs. teen focus vs. parent and teen focus	*N* = 89 at-risk youth	10–14	Post-treatment	Externalizing	Child behavior checklist – externalizing scale, self-report of tobacco use frequency	Significant increases in tobacco use frequency for teen focus and parent and teen focus groups
Forman et al. (1990)	School intervention vs. school intervention + parent intervention	*N* = 177 high-risk youth determined by school staff	Middle & high school students	Post-treatment	Externalizing	Frequency of cigarette, alcohol, and marijuana use	No significant differences between groups
Garcia-Lopez et al. (2014)	School intervention with adolescent vs. school intervention with parent training	*N* = 52 with social anxiety disorder diagnosis	13–18	Post-treatment	Internalizing	Social Anxiety Scale for Adolescents, Social Phobia and Anxiety Inventory, Brief form	Significant group differences on both measures (*ds* = 0.65 and 0.64)
Gunlicks-Stoessel and Mufson (2016)	Interpersonal psychotherapy-adolescents vs. Interpersonal psychotherapy-adolescents and parents	*N* = 15 with DSM-IV diagnosis of MDD, dysthymia, depressive disorder not otherwise specified, or adjustment disorder with depressed mood	12–17	Post-treatment	Internalizing	Children’s Depression Rating Scale-Revised	No significant differences between groups
Hardway et al. (2015)*	Adolescent intensive panic treatment vs. Adolescent intensive panic treatment + parental involvement	*N* = 57 with a primary diagnosis of panic disorder	11–18	Post-treatment	Internalizing	Children’s Depression Inventory	No significant differences between groups
Hooven et al. (2012)*	Counselors care, assess, respond, empower youth intervention vs. Counselors care, assess, respond, empower youth intervention + parent intervention	*N* = 615 identified as at-risk for suicide	High school students	Post-treatment	Internalizing	Suicide risk behaviors	Group outcomes only reported when compared to intervention as usual groups
Krinsley, 1991 dissertation	School intervention vs. school intervention + family therapy	*N* = 29 identified as at-risk for school dropout	Middle school students	Post-treatment	Externalizing	Self-reported drug and alcohol use	No significant differences between substance use at post-treatment
Lewinsohn et al. (1990)	CBT vs. CBT + parent group	*N* = 59 with DSM-III diagnosis of MDD	14–18	Post-treatment	Internalizing	Beck Depression Inventory, Center for Epidemiological Studies-Depression Scale	No significant differences between groups
Reuland and Teachman (2014)	Cognitive bias modification child-only vs. Cognitive bias modification parent-only vs. combined	*N* = 18 with diagnosis of social anxiety disorder	10–15	Post-treatment	Internalizing	Categorized as “treatment responders” based on the Social Anxiety Scale for Adolescents-Revised	No significant differences in groups based on number of “treatment responders”
Reynolds et al. (2013)	CBT vs. CBT with parent enhancement	*N* = 50 with DSM-IV diagnosis of obsessive-compulsive disorder	12–17	Post-treatment	Internalizing	Children’s Yale-Brown Obsessive Compulsion Scale	No significant difference between groups
Siqueland et al. 2005)	CBT vs. CBT + attachment based family therapy	*N* = 11with DSM-IV primary diagnosis of generalized, separation, or social anxiety disorder	12–18	Post-treatment	Internalizing	Beck Anxiety Inventory, Hamilton Anxiety Rating Scale	No significant difference between groups
Spirito et al. (2015)*	Adolescent only CBT vs. adolescent + parent CBT	*N* = 24 with DSM-IV diagnosis of major depressive disorder	11–17	Post-treatment	Internalizing	Beck Suicide Scale, Beck Depression Inventory-II	Adolescent + parent CBT participants showed greater reductions in Beck Depression Inventory scores over time (*d* = 0.67)
Waite et al. (2019)	Adolescent only CBT vs. adolescent + parent CBT	*N* = 60 with DSM-IV diagnosis of primary anxiety disorder	13–18	Post-treatment	Internalizing	Spence Children’s Anxiety Scale – parent and youth report	No significant differences between groups
Waldron et al. (2001)	Individual CBT vs. family therapy vs. individual CBT + family therapy	*N* = 114; DSM-IV diagnosis for a primary substance use disorder	13–17	4 month	Externalizing	Number of youths achieving minimal (reported use on fewer than 10% of days) versus heavy use	All three groups showed a significant change to minimal use from before treatment to 4-month follow-up
Winters et al. (2012)	Brief intervention vs. Brief intervention + parent session	*N* = 315 with DSM-IV diagnosis of substance use disorder or at least 1 or 2 dependence criteria	13–18	6 month	Externalizing	Self-report on alcohol and cannabis use days	Brief intervention + parent group evidenced significantly greater decreases in cannabis use symptoms
Wong et al. (2020)	CBT alone vs. CBT + parental involvement	*N* = 136 reporting significant anxiety symptoms	12–19	Post-treatment	Internalizing	Anxiety subscale of the Hospital Anxiety and Depression Scale, Spence Children’s Anxiety Scale	No significant differences between groups

*CBT* cognitive-behavioral therapy, *MDD* Major Depressive Disorder *DSM*  Diagnostic and Statistical Manual of Mental Disorders

*data not included in meta-analysis

**Table 2:** Characteristics of the interventions included in meta-analysis

Trial	Individual intervention		Parental involvement intervention
Bernal et al. (2019)		Twelve sessions of culturally adapted cognitive-behavioral therapy	Eight 2-hour sessions focusing on depression psychoeducation, including identifying signs of depression, family patterns that may relate to symptoms, and how to help youth cope with depression
Bogle, 2007 dissertation		An intensive after-school treatment program occurring 4 days per week for 2 h each time, targeting adolescents’ behavioral and academic problems	Four 75-minute sessions focusing on teaching behavioral management skills in an effort to address adolescents’ schooling problems
Cannabis Youth Study		Two individual sessions of motivational-enhancement treatment and ten individual cognitive-behavioral treatment sessions	Six parent psychoeducation meetings focusing on adolescent development, substance use and dependence, relapse, and family functioning
Clarke et al. (1999)		Sixteen 2-hour sessions of the adolescent coping with depression course	Eight 2-hour sessions focusing on teaching the same communication and problem-solving skills that adolescents learned in the individual adolescent coping with depression course intervention
Dishion & Andrews (1995)		Twelve 90-minute sessions teaching skills for improving emotion regulation and discussing implementation of goals at home and school	Twelve 90-minute sessions focusing on behavioral management and communication skills including role-plays and discussion of relevant issues
Forman et al. (1990)		Ten 2-hour sessions teaching coping skills, communication, and psychoeducation about substance use	Five 2-hour sessions focusing on teaching the same coping skills that adolescents are learning, as well as behavior management skills and social support
Garcia-Lopez et al. (2014)		Twelve weekly 90-minute sessions utilizing a cognitive-behavioral intervention	Five 120-minute sessions teaching psychoeducation about social anxiety and the impact of expressed emotion, as well as learning about communication and contingency management skills
Gunlicks-Stoessel and Mufson (2016)		Twelve 45-minute individual adolescent sessions of interpersonal psychotherapy	Two 45-minute individual parent sessions to obtain relevant information and teach parents’ communication and relationship skills Six 45-minute conjoint parent–adolescent sessions used to establish mutual goals, practice interpersonal skills, and discuss relapse prevention
Hardway et al. (2015)		Twenty hours total across 8 consecutive days of cognitive-behavioral therapy for panic disorder	Twenty hours total across 8 consecutive days of cognitive-behavioral therapy for panic disorder with parental involvement including psychoeducation, exposure, and skills coaching alongside adolescent through duration of treatment
Hooven et al. (2012)		Two sessions focused on assessment and motivational interviewing to target relevant risk factors and coping skills	Two sessions focused on parental assessment, suicide prevention, communication support, mood management, and problem-solving skills
Krinsley, 1991 dissertation		Intervention in the school setting, including daily meetings to discuss youths’ behavior and provide behavioral management	The number of sessions varied per family; Intervention utilized a targeted family intervention to teach problem-solving and parenting skills for the specific, unique problems families encountered
Lewinsohn et al. (1990)		Fourteen two-hour sessions of the coping with depression course intervention	Seven two-hour sessions focusing on teaching coping skills and reviewing what adolescents were learning in the individual coping with depression course intervention
Reuland and Teachman (2014)		Eight sessions of online cognitive bias modification for interpretation intervention aimed to modify adolescents’ cognitive biases specifically related to social situations	Eight sessions of online cognitive bias modification for interpretation intervention aimed to address parents’ cognitive biases related to intrusive parenting behaviors
Reynolds et al. (2013)		Fourteen individual cognitive-behavioral intervention sessions	Fourteen cognitive-behavioral intervention sessions whereby parents attended all sessions and were involved in discussing parent-related factors (e.g., accommodation)
Siqueland et al. (2005)		Sixteen sessions of individual cognitive-behavioral treatment	Sixteen sessions of cognitive-behavioral treatment in addition to attachment based family therapy. Attachment based family therapy discussed family interactions, parenting behaviors, and adolescent anxiety. The specific number of parent–adolescent vs. parent alone vs. adolescent alone sessions varied by participant
Spirito et al. (2015)		Twenty-four individual sessions of cognitive-behavioral therapy treatment	Twenty-four sessions including a compilation of individual parent and conjoint parent–adolescent sessions focused on enhancing positive communication, cognitive-behavioral therapy for parents’ depression, and skills coaching
Waite et al. (2019)		Ten 60-minute sessions followed by two booster sessions of an internet-based cognitive-behavioral intervention	Five 60-minute sessions followed by two booster sessions focusing on helping parents assist their children in acquiring and implementing cognitive-behavioral skills
Waldron et al. (2001)		Twelve 60-minute sessions including two sessions of motivational-enhancement intervention and ten sessions of cognitive-behavioral treatment	Twelve 60-minute sessions of a systems-oriented treatment aimed at targeting unhelpful family patterns that relate to adolescents’ substance use problems
Winters et al. (2012)		Two 60-minute individual sessions focusing on motivational interviewing and identifying and following up on goals for change	One 60-minute session using motivational interviewing to discuss adolescent substance use and related parenting skills
Wong et al. (2020)		Eight 2-hour sessions following the original coping cat program culturally adapted for Chinese adolescents	Five 2-hour psychoeducation sessions including discussion of parental anxiety, accommodation, and exposure coaching.

### Quantitative Findings

Summary statistics suggested that interventions that involved parents in treatment had a significantly greater impact on adolescent psychopathology when compared to interventions that targeted adolescents alone (*g* = − 0.18, *p* = .002, 95% CI [− 0.30, − 0.07]). While statistically significant, the overall effect size was small. Effect size data from each individual study are presented in Table 3. Additional sensitivity analyses completed involved calculating findings when each individual study was removed one at a time from overall analyses. Results remained significant when each individual study was removed. Results were examined further with symptom type (internalizing or externalizing) included as a moderator. The significant intervention difference remained for externalizing (*g* = − 0.20, *p* = .01, 95% CI [− 0.35, − 0.05], *k* = 7) but was not significant for internalizing psychopathology (*g* = − 0.15, *p* = .11, 95% CI [− 0.34, 0.03], *k* = 10). The difference between the effect sizes for externalizing (− 0.20) and internalizing (− 0.15) symptoms was not statistically significant (*p* = .70). Outcome type, including diagnostic, dimensional, or frequency (e.g., number of alcohol use days) was also a significant moderator of study findings. Specifically, findings remained significant for frequency outcomes (*g* = − 0.23, *p* = .01, 95% CI [− 0.42, − 0.05], *k* = 3) but were no longer significant for diagnostic (*g* = − 0.24, *p* = .08, 95% CI [− 0.49, 0.02], *k* = 4) or dimensional outcomes (*g* = − 0.12, *p* = .27, 95% CI [− 0.32, − 0.09], *k* = 9). Similar to symptom type, the differences between effect sizes were not statistically significant (*p* = .73). Number of sessions parents was involved in, outcome assessment timeframe, age, and study quality did not significantly moderate study findings (*ps * > 0.05).

**Table 3:**
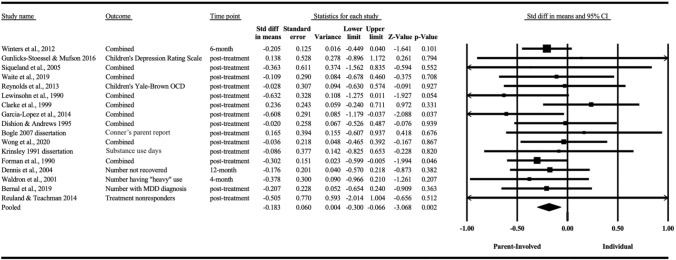
Quantitative findings

**Fig. 2:**
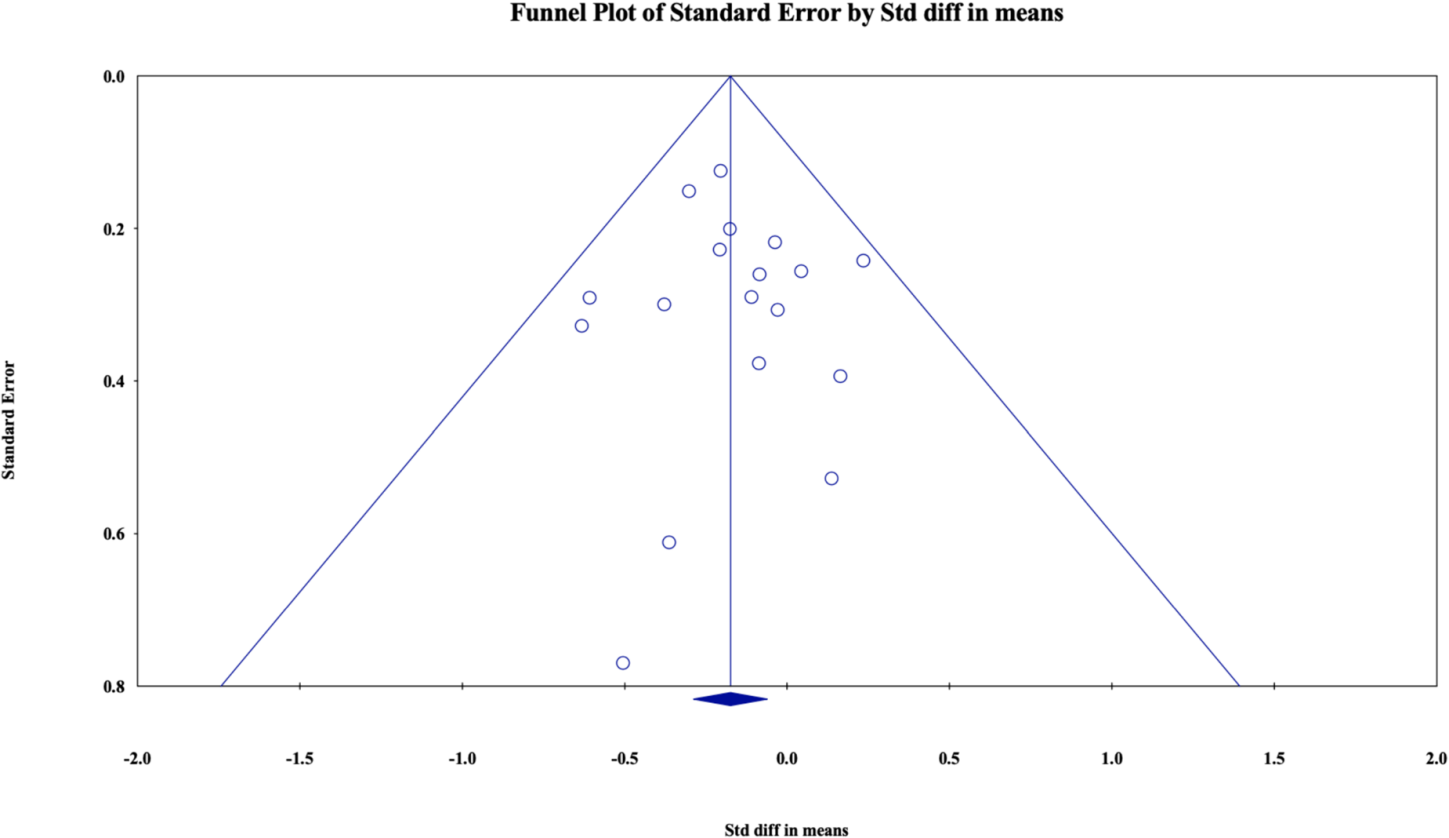
Funnel plot from meta-analysis

The *Q*-test for heterogeneity was not significant (*Q*-value = 10.72, *p* = .83) and less than the degrees of freedom (*df* = 16). As such, the amount of between-study variance was less than what we would expect based on sampling error alone. In addition, as a result, *I*^2^ is equal to 0%, suggesting that all variance in observed effect sizes was due to sampling error, as opposed to variance in true effects (Borenstein, 2019). This means no clinically significant heterogeneity among true effect sizes. Visually inspecting funnel plots showed minimal evidence of publication bias. The funnel plot is presented in Fig. 2. Egger’s test was conducted and showed a non-significant result (B_0_ = 0.11, *p* = .83), suggesting no significant evidence of publication bias.

## Discussion

The primary aim of this meta-analysis was to examine whether parental involvement in treatment adds additional benefit beyond individual psychological interventions for adolescents. Parent involvement typically occurs for interventions with pre-adolescent children due to children’s dependency on parents for support, but parental involvement may also play an important role in interventions among adolescents. Given that current interventions are not effective for all youth (Weisz et al., 2017), including parents in treatment of adolescents may improve outcomes.

Findings from the current study suggest that interventions involving parents generated significantly greater impact on psychopathology than matched interventions that only involve adolescents. Importantly, the effect size of this difference was small (*g* = − 0.18) but represents an effect over and above individual interventions. Moreover, other results including symptom and outcome type as a moderator suggest that the advantage for parental involvement was significant for externalizing (*g* = − 0.20) but not for internalizing (*g* = − 0.15) problems, as well as significant for frequency (*g* = − 0.23), but not for diagnostic (*g* = − 0.24) or dimensional outcomes (*g* = − 0.11). Within moderator analyses, effect sizes were not statistically significantly different from each other. Results highlight the potential benefits of adding parent-based components to psychological interventions for adolescent externalizing problems.

### Quantitative Findings

As this is the first meta-analysis to the authors’ knowledge that has examined parent-involved interventions across disorder groups with an exclusively adolescent sample, a discussion of findings includes data from previous reviews conducted in both children and adolescents. There continues to be some differences of opinions as to what constitutes the beginning of adolescence, so the age ranges of samples are reported when possible to increase clarity. Findings from the current meta-analysis replicate some (Dippel et al., 2022; Dowell & Ogles, 2010), but not other (Peris et al., 2021; Vermeulen-Smit et al., 2015), results from such reviews. One prior meta-analysis among youth ages 3–18 years old examined comparisons of individual child treatment to combined parent–child/family treatment. This meta-analysis found significant benefits for parent–child/family interventions (*d* = 0.27), above and beyond individual treatments (Dowell & Ogles, 2010). However, unlike in the current paper, the nature of presenting problems, indicated as “internalizing,” “externalizing,” or “other”, did not moderate the effect of parent involvement on outcomes. Discrepancies in findings between the current study and past research are also evident in disorder-specific meta-analytic findings. For example, while one meta-analysis found a small, significant effect of family-involved interventions for children and adolescents ages 3–18 with depression (Dippel et al., 2022), other similar meta-analyses among youth ages 6–18 with anxiety (Peris et al., 2021; Thulin et al., 2014) and adolescents with substance use (Vermeulen-Smit et al., 2015) did not demonstrate such an effect. These meta-analyses used some similar inclusion criteria as in the current paper but examined outcomes with both a broader age range (Peris et al., 2021; Thulin et al., 2014) and broader set of comparison conditions (Dippel et al., 2022; Vermeulen-Smit et al., 2015). While prior research has not consistently found benefits to parent involvement, the results from the current meta-analysis may relate to unique features of the current study.

The findings from the current meta-analysis are also informative as they relate to work with younger child samples. Past work in this age group finds benefit of parental involvement for the treatment of externalizing more so than internalizing problems (Buchanan-Pascall et al., 2018; Mingebach et al., 2018). For example, one meta-analysis reported a significant effect size for parent training on both externalizing and internalizing problems among 4–12-year-old youth (Buchanan-Pascall et al., 2018). However, the effect size for internalizing problems (*g* = − 0.18) was smaller than for externalizing problems (*g* = − 0.38). When considered in connection to findings from the current meta-analysis, parent involvement appears to produce more consistent benefits for externalizing as opposed to internalizing psychopathology.

### Methodological Considerations

Several aspects of study designs and methodologies warrant consideration in interpreting the findings from this meta-analysis. For example, extant research on pediatric anxiety disorders discusses factors that could attenuate the effect of parent involvement in youth interventions for psychopathology (Breinholst et al., 2012; Peris et al., 2021; Silverman et al., 2022). Specifically, greater emphasis might be placed on measuring and including family-level outcome variables to fully capture the impact of parent involvement (Breinholst et al., 2012; Peris et al., 2021). These insights may also be relevant to adolescent focused work. One study in particular included in the meta-analysis randomized Puerto Rican adolescents with depression to receive a culturally adapted treatment including either 12 individual sessions of CBT or 12 CBT sessions and an 8 session parent psychoeducation group intervention (Bernal et al., 2019). While results found no difference between treatment conditions on adolescent depression, there were significant group differences on family-level variables, including familism and family emotional involvement. Overall findings from adolescent and parent interventions may differ based upon the type of outcome assessed. Defining a successful trial might depend upon which outcome variables are included and what is the hypothesized mechanism(s) for change.

In addition to including family-level variables, it is also important to examine both youth and parental moderators (Garcia-Lopez et al., 2014). Garcia-Lopez et al. (2014) reported on a trial whereby families were randomly assigned to either an individual or family school-based CBT intervention for adolescents with social anxiety disorder and parents high in expressed emotion. Interestingly, parent expressed emotion status moderated findings, suggesting that adolescents whose parents *changed* status from high to low expressed emotion had significantly lower anxiety scores those than those whose parents stayed at high expressed emotion. Findings highlight the importance of assessing parental moderators, such as parental expressed emotion or psychopathology, as they could influence efficacy of treatment when parents are involved.

Finally, ways in which parents are involved may differentially affect youth psychopathology (Peris et al., 2021; Silverman et al., 2022). For example, parents may be included as co-therapists when youths’ symptoms are the main treatment target (Spence et al., 2000) or co-clients when their symptoms are targeted in addition to their child’s (Spirito et al., 2015). Some interventions involved parents within the same session (Gunlicks-Stoessel & Mufson, 2016) and others utilize separate parent sessions altogether (Bernal et al., 2019). Further, some interventions for anxiety in particular have utilized parent-only interventions (Jewell et al., 2022), such as SPACE (Lebowitz et al., 2020). Results from these trials suggest that these may be as effective as individual interventions for some disorders. Additional care should be taken into how parents are involved, as well as how this involvement is being assessed, to understand the full benefit of parent-involved interventions with adolescents.

There may be other explanations as to why parental involvement did not add benefit over and above individual treatment for internalizing disorders. There may be less of a difference between the efficacy of individual and parent interventions because individual interventions for internalizing psychopathology, specifically anxiety, on their own generate a relatively large treatment effect. This compares to individual interventions for externalizing psychopathology (Farmer et al., 2002; Weisz et al., 2004, 2017), which are less often utilized and generate smaller effects. This possibility is reinforced by examining differences in effect sizes for individual interventions included in the meta-analysis. These studies show that effect sizes for individual treatments for anxiety (Garcia-Lopez et al., 2014; Reynolds et al., 2013) are larger than those for individual treatments for substance use (Barrett et al., 2001; Winters et al., 2012). Additional work is needed to confirm whether parents should be included in adolescent interventions differently based upon disorder type.

Another explanation as to why there is not a significant difference for individual vs. parent-involved interventions for internalizing problems may relate to the differing levels of symptom severity among youth in the included studies. Specifically, all but one article (Waldron et al., 2001) examining externalizing psychopathology included youth with some subthreshold symptoms or at-risk behaviors in addition to those who meet full criteria for a diagnosis. This is in contrast to included articles examining internalizing psychopathology whereby all but one (Wong et al., 2020) of the articles included in the meta-analysis required youth to meet criteria for a diagnosis. Youth in the papers with internalizing problems likely had more severe levels of problems. These differing levels of risk might suggest that parental involvement generates greater benefit for those with subthreshold problems. This is further reinforced by findings in universal parent-involved interventions (Schinke et al., 2004) showing a benefit to involving parents in interventions even when youth have lower levels of symptoms. There is a need for more research among adolescents with differing levels of symptomology to confirm how disorder severity may relate to parent-involved treatment efficacy.

It is also possible that there is something specific to internalizing disorders during adolescence may make parental involvement more challenging. Internalizing when compared to externalizing problems may be less visible to parents, as adolescents may be more hesitant to share what they are thinking and feeling. This is important when thinking about how outcomes can differ based upon the type of informant (Weisz et al., 2017). Given that adolescence can be associated with decreases in parental monitoring, as well as increasing stress in the parent–child relationship, parents and youths may have differing perspectives on the success of treatment. A surprisingly small (*k* = 5) number of studies in the current meta-analysis included parental reports of adolescent symptoms. Future work should aim to assess whether results may differ based upon parent versus child report.

In addition to symptom type, outcome type was also a significant moderator of findings, such that interventions involving parents were significantly more beneficial when frequency-based outcomes (e.g., number of alcohol use days) were assessed. This difference was no longer significant for diagnostic or dimensional outcomes. Of note, only three studies included frequency-based outcomes, all of which assessed substance use outcomes. Two of these studies also had the largest sample size of included work (Forman et al., 1990; Winters et al., 2012). Finally, although effect sizes were similar for frequency-based (*k* = 3, *g* = − 0.23; *p* = .01) and diagnostic (*k* = 3, *g* = − 0.24; *p* = .08) outcomes, only frequency-based outcomes yielded a significant benefit for parent-involved interventions. The smaller variance for frequency-based (*σ*^2^ = 0.009) as compared to diagnostic (*σ*^2^ = 0.018) outcomes may help to explain why the former was significant. These considerations suggest that replication is warranted to confirm the significance of frequency-based outcomes.

### Strengths, Limitations, and Future Directions

The current study has several strengths, including the novel focus on involving parents in psychological interventions for adolescents with a variety of psychiatric problems. Prior meta-analytic work examining efficacy of parental involvement has grouped children and adolescents together (Dowell & Ogles, 2010). This is problematic because adolescence represents a developmental time period with a unique set of psychosocial stressors and challenges (Steinberg & Morris, 2001). The methods used to involve parents in the treatment of adolescents are likely to differ from the methods used in the treatment of children. With such differences, combining studies in children and adolescents could be inappropriate. As such, a focus on efficacy studies in this age group, separate from childhood, is critical. Additionally, the choice of inclusion criteria in the current meta-analysis successfully balances heterogeneity and thoroughness. This helps to assess the benefit of parental involvement over and above individual treatment without evidence of significant heterogeneity or publication bias. The lack of significant heterogeneity likely resulted from the limited variability in diagnoses captured by the inclusion criteria in existing research, as well as the specificity of the included study design and the overall small number of included studies. On the one hand, many meta-analyses do find heterogeneity, even with a relatively small number of studies. Hence, it could be viewed as surprising to observe homogeneity. On the other hand, other prior met-analyses (e.g., Thulin et al., 2014; Vermeulen-Smit et al., 2015) also failed to find heterogeneity, suggesting some replicability in this pattern.

In addition to study strengths, there are also several limitations that should be noted. One limitation is that quantitative analyses excluded three eligible studies (Hardway et al., 2015; Hooven et al., 2012; Spirito et al., 2015) due to lack of access to relevant data to calculate effect sizes. It is worth noting that two (Hardway et al., 2015; Spirito et al., 2015) out of three of these studies did not find a significant difference between individual and parent-involved interventions. Further, all three of these studies assessed interventions’ impact on internalizing psychopathology; the significant impact of parent involvement for externalizing versus internalizing problems would be unaffected and could remain significant even if these three studies were included in analyses. Additional limitations include the relatively moderate number of studies in the meta-analysis (*k* = 20) and the overall small effect size (*g* = − 0.18), which even though it is statistically significant, limits clinical applicability. The included studies involved parents in different ways (e.g., psychoeducation, co-therapist) and lack of sufficient variability in included studies prohibited exploring the differential impact of this. Lastly, included studies for the current meta-analysis only captured depression, anxiety, obsessive-compulsive disorder, and substance-use-disorder diagnoses. It is surprising that this relatively narrow group of disorders was captured. This precludes the generalizability of findings to these other diagnoses. Many possibilities could account for our failure to identify conditions beyond this selected set of disorders. For example, our review focused narrowly on relatively rigorous clinical trials, which are expensive to implement. Funding priorities could contribute to this limitation, prioritizing research on the conditions identified in our review. More research with the included design is needed to assess the potential benefit of parent-involved across a wider range of diagnoses.

The findings of this review are somewhat limited regarding conclusions that can be drawn about treatment mechanisms, as mechanisms may differ based upon disorder type. For example, family accommodation is especially important for understanding trajectories of anxiety disorders (Lebowitz et al., 2016); family conflict relates particularly closely to adolescent depression and obsessive-compulsive disorder (Rice et al., 2006; Waters & Barrett, 2000); other parenting behaviors are critical for altering youth depression risk (Compas et al., 2015); and decreased parental monitoring is associated with adolescent substance use problems (Rusby et al., 2018). Research on treatment mechanisms for adolescent therapy more broadly, let alone with parent-involved interventions, remains preliminary (e.g., Kazdin, 2007; Taubner et al., 2023). As future work clarifies when and how parents should be involved in adolescent treatment, additional work will be needed to understand mechanisms of such successful treatments.

These limitations generate pathways for future research. More recent studies in both children and adolescents suggest the possibility of randomizing parents to different types of parent-involved interventions (Kagan et al., 2022; Manassis et al., 2014; Peris et al., 2017; Silverman et al., 2022). In one randomized controlled trial, 8–17-year-old youth with a primary diagnosis of obsessive-compulsive disorder and poor family functioning were randomized to receive either 12 sessions of individual CBT with weekly parent psychoeducation or the same 12 sessions of individual CBT with 6 sessions of family therapy (Peris et al., 2017). When compared to the parent psychoeducation condition, the family therapy condition evidenced better remission rates, reductions in functional impairment, and improvements in family cohesion. A similar intervention trial randomized parents to receive different CBT interventions. In this study, 7–16-year-old youth with a primary anxiety disorder diagnosis were randomized to either individual CBT, CBT targeting parents’ reinforcement skills, or CBT targeting parents’ relationship skills (Silverman et al., 2022). At post-treatment, youth in the two CBT parent conditions evidenced lower anxiety scores than those in individual CBT. Results suggest specificity in parenting outcomes, as families assigned to the reinforcement skills condition showed less negative reinforcement when compared to the other two conditions. The novel approach in these studies compared different ways in which parents may be involved in interventions. Findings from both of these trials and others (Manassis et al., 2014) suggest that different types of parental involvement may differentially impact parent and youth outcomes.

Future studies might also consider how family circumstances, treatment setting, clinician type, and experiences of adversity may impact parent involvement in adolescent interventions (Baker-Ericzén et al., 2013). Youth who have experienced early adversity are at greater risk for developing later psychopathology (McLaughlin et al., 2019) and have greater difficulty accessing evidence-based care (Schweer-Collins & Lanier, 2021). Involving parents from these families in interventions brings challenges. For example, parents living in poverty experience chronic stress (Ceballo & McLoyd, 2002), and the demands of work, other children, and lack of resources may limit their availability to engage in therapy with their child. Some circumstances may even prevent parents from any involvement, such as if parents are perpetrators of abuse and youth have been removed from their parents’ homes. Future research may aim to explore novel ways, such as using telehealth or separate parent–child sessions, to accommodate these concerns and increase the accessibility of parent-involved treatment.

Ultimately, adolescence is a unique developmental time period for building autonomy and independence. With these changes, parents continue to play a critical, protective role (Steinberg & Morris, 2001). Adolescents are at greater risk for the development of psychopathology and current interventions are not effective for all youth (Weisz et al., 2017). Findings from the current paper highlight the importance of considering parental involvement to improve treatment efficacy.
